# Oxygen Mapping of Melanoma Spheroids using Small Molecule Platinum Probe and Phosphorescence Lifetime Imaging Microscopy

**DOI:** 10.1038/s41598-017-11153-9

**Published:** 2017-09-06

**Authors:** Ahtasham Raza, Helen E. Colley, Elizabeth Baggaley, Igor V. Sazanovich, Nicola H. Green, Julia A. Weinstein, Stanley W. Botchway, Sheila MacNeil, John W. Haycock

**Affiliations:** 1 Materials Science & Engineering, University of Sheffield, Sheffield, S3 7HQ UK; 2 School of Clinical Dentistry, University of Sheffield, Sheffield, S10 2TA UK; 3 Department of Chemistry, University of Sheffield, Sheffield, S3 7HF UK; 4 Research Complex at Harwell (CLF), STFC Rutherford Appleton Laboratory, Oxford, OX11 0QX UK

## Abstract

Solid tumours display varied oxygen levels and this characteristic can be exploited to develop new diagnostic tools to determine and exploit these variations. Oxygen is an efficient quencher of emission of many phosphorescent compounds, thus oxygen concentration could in many cases be derived directly from relative emission intensity and lifetime. In this study, we extend our previous work on phosphorescent, low molecular weight platinum(II) complex as an oxygen sensing probe to study the variation in oxygen concentration in a viable multicellular 3D human tumour model. The data shows one of the first examples of non-invasive, real-time oxygen mapping across a melanoma tumour spheroid using one-photon phosphorescence lifetime imaging microscopy (PLIM) and a small molecule oxygen sensitive probe. These measurements were quantitative and enabled real time oxygen mapping with high spatial resolution. This combination presents as a valuable tool for optical detection of both physiological and pathological oxygen levels in a live tissue mass and we suggest has the potential for broader clinical application.

## Introduction

The use of metal complexes as dyes and probes for emission-based cellular imaging has developed into a vibrant area of research over the past decade^1–3^. This emerging class of probes offers photo-physical properties that differ to a range of commercially available organic probes, and is enabling a new range of technologies to be explored. Notably, recent advances in optics and electronics, combined with the long emission lifetimes typical for transition metal complexes (from hundreds of nanoseconds to microseconds), has resulted in the emergence of multiphoton microsecond lifetime mapping techniques, such as Phosphorescence Lifetime Imaging Microscopy (PLIM)^4, 5^ and Time-Resolved Emission Imaging Microscopy (TREM)^4^.

Luminescent transition metal complexes typically emit from a triplet excited state. Although transitions between states of a different spin are formally forbidden, intersystem crossing to the triplet state and subsequent relaxation to the ground state via phosphorescence are facilitated in transition metal complexes by the high spin orbit coupling constant associated with the heavy metal atom. The forbidden nature of the phosphorescence transition, results in a slow rate of emission; hence phosphorescence lifetimes are typically the order of hundreds of nanoseconds to microseconds. It is well documented that molecular oxygen quenches such triplet emitters and that the rate of quenching is dependent on oxygen concentration^5–10^. One application of phosphorescent emitters in biological imaging is in oxygen detection, where more sensitive, non-invasive methods are in demand. The combination of phosphorescence quenching and high-resolution lifetime imaging is a powerful approach for non-invasive, real-time hypoxia detection and oxygen quantification.

Oxygen quantification in biological systems is primarily focused on the use of large platinum and palladium porphyrins. These compounds display long emission lifetimes (typically 40–60 µs), which typically decreases upon exposure to oxygen. They have been successfully used for measuring bulk intracellular oxygen concentrations in a range of models. Thus they have been used in an intensity-based, high-throughput method utilizing a time-resolved fluorescence plate reader^5–7^; in determining oxygen gradients across 2D monolayers, 3D neurospheres and spheroids using a combination of single photon time correlated single photon counting (TCSPC) and PLIM; in measuring *in-vivo* oxygen concentration changes in the blood stream of live animals, using bespoke multi-photon microscope^10–13^.

Efficient cellular uptake can be problematic with porphyrins, due to their large size. For some applications, such as intra-cellular and blood oxygen concentrations, this is a positive attribute as it prevents the porphyrin from sequestering into the surrounding cells or tissue. For intercellular oxygen determination and penetration into larger or deeper samples, it is a major drawback. This problem has been overcome in some cases by the addition of cell-penetrating moieties, specific cell markers or encapsulation in nanoparticles, however, these systems are complex and require long multi-step syntheses.

Phosphorescence quenching offers a direct, real-time and quantitative method for oxygen determination^14–16^. To obtain a detailed relationship between cellular location of the probe and its emission lifetime (and therefore oxygen concentration) excellent spatial and temporal resolution is required. Intensity based imaging using wide field illumination will only give a general picture of the oxygen concentration as the intensity information will be averaged in the ‘z’ direction. Furthermore, intensity based images can be affected by background emission, probe concentration, imaging parameters and photobleaching. Lifetime mapping using a point-by-point scanning method, such as that used in confocal imaging, with time correlated single photon counting, offers a more accurate method that circumvents many of the drawbacks associated with intensity imaging. Yet there is a hurdle: the longer the lifetime of the O_2_ probe, the longer the image acquisition time. To establish a lifetime decay at each pixel (in a typical 256 × 256 array), with a good signal-to-noise, the microscope scan head must remain at each pixel for a period of time that is 5 to 6 times longer than the emission lifetime of the probe, and over tens or hundreds of frame scans. Therefore, porphyrins with very long emission lifetimes require very long acquisition times, over which sample drifting can be a problem. One solution is to sacrifice spatial resolution by using a smaller number of larger pixels. Alternatively, using an oxygen responsive probe with a shorter emission lifetime can reduce sampling time.

Small molecule transition metal complexes with emission lifetimes on the order of hundreds of nanoseconds to microseconds provide an alternative that requires less of a trade-off between resolution and speed, with simpler synthetic procedures. Some transition metal complexes can penetrate living cells without the need for elaborate synthetic modification and in some cases demonstrate specificity to particular organelles, which can be desirable when investigating oxygen changes as a response to external stimuli.

**Figure 1:**
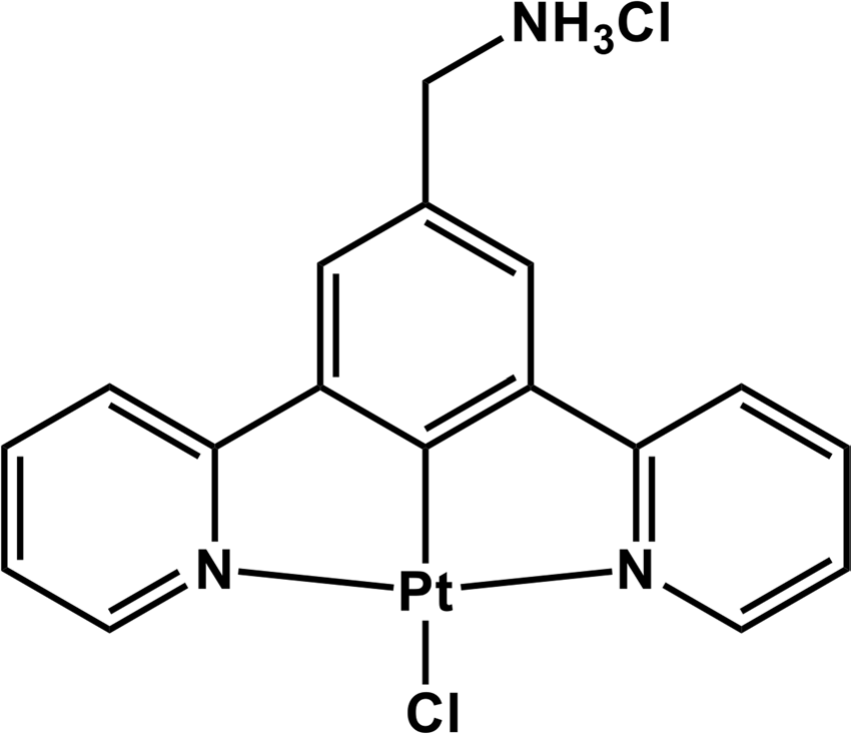
Structure of PtL^s^Cl.

We have previously reported on the application of a series of small luminescent platinum (II) complexes PtL^n^Cl for *in vitro* live cell imaging and two-photon lifetime mapping using TCSPC^17, 18^. This family of complexes (where L is a cyclometalating 3-di(2-pyridyl)benzene) based ligand) (Fig. 1) exhibit microsecond emission lifetimes (τ_0_ = 7 µs in CH_2_Cl_2_), high emission quantum yields (c.a. 70%)^19^, excellent photostability and low cytotoxicity. Previous studies have investigated the mechanism of entry across human fibroblast and melanoma cells *in vitro*, and report on properties that enable passage across intact cell membranes via diffusion after incubation with PtL^n^Cl for 5 minutes, permitting imaging thereafter^17^. Moreover, these PtL^n^Cl complexes have a relatively short synthetic pathway, which can be accelerated by microwave-assisted methods^20^. We have already demonstrated that the emission lifetime of complexes of this type varies with exposure to oxygen inside living cells (CHO cell cytoplasm from 95% N_2_/5% Co_2_ from 1.49 µs to 6 µs^4, 18^). Cytosol staining appears uniform under two-photon excitation and exhibits the lowest level of protection from collisional quenching, making these complexes an excellent choice for oxygen detection via phosphorescence quenching. Another key advantage of such small Pt(II) molecules is their diffusion controlled intracellular accumulation without an active intake pathway, which makes them ideal as probes for experimental systems such as 3D tumour spheroid models.

Multicellular tumour spheroids (MCTS) involve culturing cancer cells in a non-adherent environment, which consequently stimulates them to adhere with one another^21, 22^. These solid aggregates resemble an avascular tumour^23^. The model provides an assay to study the tumour microenvironment where the outermost layers of cells receive nutrients by diffusion and discard waste products into culture medium. However, the innermost cells outgrow the natural diffusion range of nutrients, oxygen and waste products, leading to formation of hypoxia and necrosis within the inner ‘core’ areas. The extent of these features can be controlled experimentally as they are determined by the type of cells in culture, the size of the MCTS, time in culture, together with the proliferation rate of the cancer cells in question.

It has been established that not all cell types have the ability to form spheroids, and some cell types form irregular shaped aggregates rather than spheroids. The heterogeneity of cells and the metabolic gradients (including oxygen, glucose, ATP) become apparent in spheroids only when they are larger than 150 µm. The appearance of a necrotic and hypoxic central region becomes apparent only after a diameter of ~500 µm^23–25^. Therefore, 3D MCTS models can be exploited as a structural model with a necrotic central core due to: (i) deficiency of nutrients and metabolites; (ii) variation in oxygen concentration; (iii) accumulation of waste products and (iv) low pH. These are some characteristics related to the phenomena seen *in vivo* of a solid tumour over a distance >100 µm from a blood supply^26, 27^.

Extending upon previous studies using small molecule Pt dyes^4, 17, 18^, herein we use a human melanoma multi-cellular tumor spheroid model in a longitudinal study and investigate the small molecule Pt dye for variations in oxygen tension. This progresses previous single melanoma cell reports to an organoid study of physiological context. We demonstrate the first example of a small-molecule platinum (II) complex for oxygen mapping using one-photon phosphorescence lifetime imaging microscopy, which provides a non-invasive imaging modality for oxygen distribution across live human melanoma tumour spheroids at various stages of development.

## Results

To mimic solid tumour conditions, we used a melanoma multi-cellular tumour spheroid model (MCTS). MCTS models exhibit characteristics of an *in vivo* solid tumour, such as, heterogeneous proliferative and necrotic cells and varied metabolic gradients of oxygen, nutrients and waste product^23, 28^. These tumour microenvironments exhibit three distinct regions of oxygenation^28–30^, simulating the oxygen gradient found across a solid tumour; where the majority of tumour cells located close to a blood vessel are proliferative while the inner area (far from the blood vessel) is made up of hypoxic and anoxia cells^29^. Using an *in vitro* melanoma C8161 tumour spheroid model (between 100 µm and 1000 µm in diameter) we successfully characterized these three physically distinct regions: (a) a proliferative viable region of human melanoma cells, (b) an inner hypoxic region and (c) a central necrotic core, using complementary methods of light microscopy, histology, immunohistochemistry and phosphorescence lifetime mapping.

### MCTS growth

**Figure 2:**
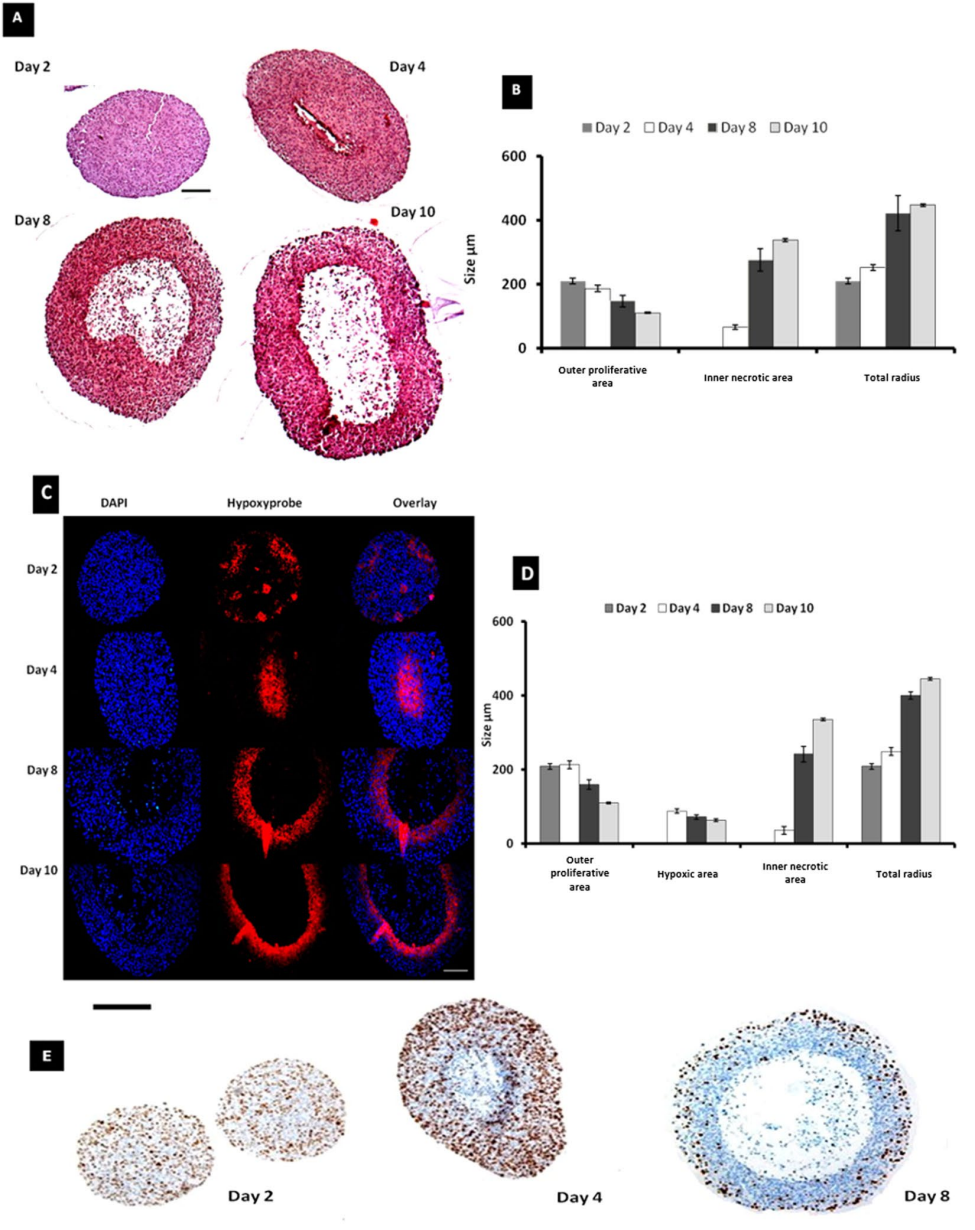
Morphology of C8161 MCTS. (**A**) H&E of human melanoma C8161 MCTS after 2, 4, 8 and 10 days in culture (Magnification 10X, scale bar = 100 μm). (**B**) Measurement of outer and inner necrotic area of MCTS (n = 10, mean ± S.E). (**C**) Immuno-labelling of melanoma C8161 MCTS by Hypoxyprobe™ (red), counterstained with DAPI (blue) (Magnification 20X, scale bar = 100 μm). (**D**) Measurement of outer area, hypoxic area and central necrotic area of MCTS with respect to time in culture (n = 10, mean ± S.E). (**E**) C8161 MCTS stained with anti-Ki67 to show proliferating cells (brown) counterstained with haematoxylin (Magnification 10X, scale bar = 200 µm).

Three different human melanoma cell lines (A375-SM, HBL and C8161) were initially investigated for their ability to form multi-cellular tumour spheroids (MCTS) *in vitro*, using an established liquid overlay technique^21^. C8161 melanoma cells were the most successful, forming compact MCTS within first 1–2 days of culture with a very high yield of 90 to 100%. A seeding density of 12,000 cells was selected as the optimal starting point for the C8161 MCTS model (from a range tested across 750–24,000 (Figure SI1). The size (diameter) of melanoma MCTS determined over a 10-day growth period by phase contrast microscopy (Figure SI1) showed initial exponential growth, from 553 µm ± 25.5 (n = 66) at day 4 to 952 µm ± 48.5 (n = 66) at day 10, with a volume doubling time of 8 days. No stationary phase was observed under these conditions. An outer region clearly comprised of a cellular mass and a central region devoid of cells was evident after 4 days in culture, by light microscopy (Figure SI1). This was not the case seen with a lower seeding densities (7,500 and 1,500), where an exponential growth in early spheroid development was observed followed by a later stationary phase at 8 to 10 days. MCTS cultured for 5 different time periods (2, 4, 8 and 10 days) were fixed and processed for histology. Haemotoxylin & eosin staining (Fig. 2A,B) revealed a uniformly stained cell mass at 2 days, and a central area visible after 4 days, which did not take up H&E stain and which increased in size over time. This lack of staining was attributed to a reduction in cellular mass in the central core of the spheroid, and arises from the formation of debris following cell death. It was therefore referred to as a necrotic area. This observation was consistent with Ki-67 staining (Fig. 2E), which identified proliferating cells (in brown) across the entire spheroid at day 2, but was restricted to the edges of spheroids by day 8. Spheroid formation at day 2 was therefore predominantly via cell division due to the extent of Ki67 uniformity, but may include a degree of aggregation or coalescence at this early stage.

The radius of the unstained necrotic region increased in size from 65.6 µm (±22.5) on day 4 to 338.2 µm (±11.8 µm) (n = 10) on day 10, whereas the proliferative area reduced in size from 186.4 µm (±31.3) to 109.7 µm (±5.7) µm (Fig. 2B). After 8 to 10 days in culture, the cellular mass was not homogenous across the spheroids. Cells near to the periphery were loosely attached and had a large extracellular space compared with medially located cells, which were very tightly packed. The core was observed to contain a very small number of cells with mostly cell debris. The results clearly showed that formation of an un-stained central core was dependent on the size of the spheroid and the time in culture.

### Oxygen profiling

#### End-point detection

Hypoxic regions within MCTS across different growth stages were detected using pimonidazole hydrochloride with immuno-labelling of protein adducts of reductively activated pimonidazole as evidence of hypoxia. A commercially available kit (Hypoxyprobe™) was employed using the pimonadizole as an oxygen marker that binds only to cells with an oxygen saturation less than pO_2_ of 10mm Hg at 37 °C^31^. In MCTS at day 4 a central hypoxic area was observed (red, Fig. 2B, radius = 87 ± 18 µm), which developed in to a ring-like structure, surrounding the central un-stained core after 8 and 10 days (radius = 159 ± 36 µm (8 days) and 109 ± 6 µm (10 days), respectively). Evaluation of MCTS using Hypoxyprobe™, Ki-67 and haemotoxylin & eosin staining all revealed the presence of a central core (by day 8) that exhibited negative staining in this region. This was attributed to the presence of necrosis, consisting mostly of cellular debris as a result of deprivation of inwardly diffusing and transported nutrients. Cumulatively, the data showed that C8161 melanoma MCTS grown between 4 and 8 days and greater than 500 µm in size exhibit three distinct regions: an inner central necrotic region, an intermediate hypoxic region and an outer proliferative region. Hypoxyprobe™ was not much expressed in the necrotic area of the spheroid merely due to presence of less or no cells in necrotic area, which was consistent with previous data reported for cervix carcinoma^31, 32^. It is believed that the Hypoxyprobe™ positive regions are localized to a viable tumour, conversely necrotic cells are unable to metabolize the marker and were therefore do not express immunoreactive sites. However, the Hypoxyprobe™ method, while validated in a number of clinical and experimental studies^31^, is only able to detect areas of hypoxia within a spheroid as an indirect endpoint method, and lacks quantitative analysis. To obtain non-invasive, real-time O_2_ quantification within a spheroid model, we therefore correlated the data obtained above, by histology and hypoxia, with phosphorescence lifetime mapping using a small platinum (II) molecule O_2_ sensor and PLIM^11, 12, 33, 34^.

#### Real-time mapping of tumour growth using Pt-label and PLIM

To map the oxygen concentration across the width and depth of the C8161 human melanoma MCTS using PtL^s^Cl and lifetime mapping, we first assessed the penetrative ability of the PtL^s^Cl in to the spheroids by steady-state microscopy under two-photon excitation (λ_ex_ = 800 nm and λ_em_ = 500–550). Previous studies (and this work, see SI) on 2D cell monolayers demonstrated that PtL^s^Cl is rapidly taken up by living cells after just 5 minutes incubation at 100 µM. In contrast, at the same concentration, we found that PtL^s^Cl required 12 hours at 37 °C to completely penetrate though a C8161 melanoma spheroid of <500 µm in diameter. (Higher concentrations of label (up to 1000 μM) for 5 minutes incubation did not penetrate significantly beyond the outer cell layer, and was similar to the depth observed using 100 μM). PtL^s^Cl uptake and distribution was uniform across spheroids of different sizes at different growth stages (Figure SI2A–D). Penetration though a typical spheroid was confirmed via emission spectra and steady-state two-photon imaging (Figure S2E) at a range of depths.

The emission lifetime of PtL^s^Cl in solution is significantly reduced in the presence of oxygen (from τ = 5.4 µs without oxygen, to τ_0_ = 0.17 µs in aerated DMF [4]). Previous studies (and this work, see SI) also show that the probe remains sensitive to oxygen when inside a living cell; exemplified by the variation in emission lifetime across a single cell where τ_nucleoli_ ~ 7 µs > τ_nucleus_ ~ 5.6 µs > τ_cytosol_ ~ 4.0 µs (Figure SI3) as a result of different binding environments offering different levels of protection from collisional quenching with dissolved oxygen. Using these data, we investigated the emission lifetime of PtL^s^Cl under PLIM in the melanoma MCTS model.

**Figure 3:**
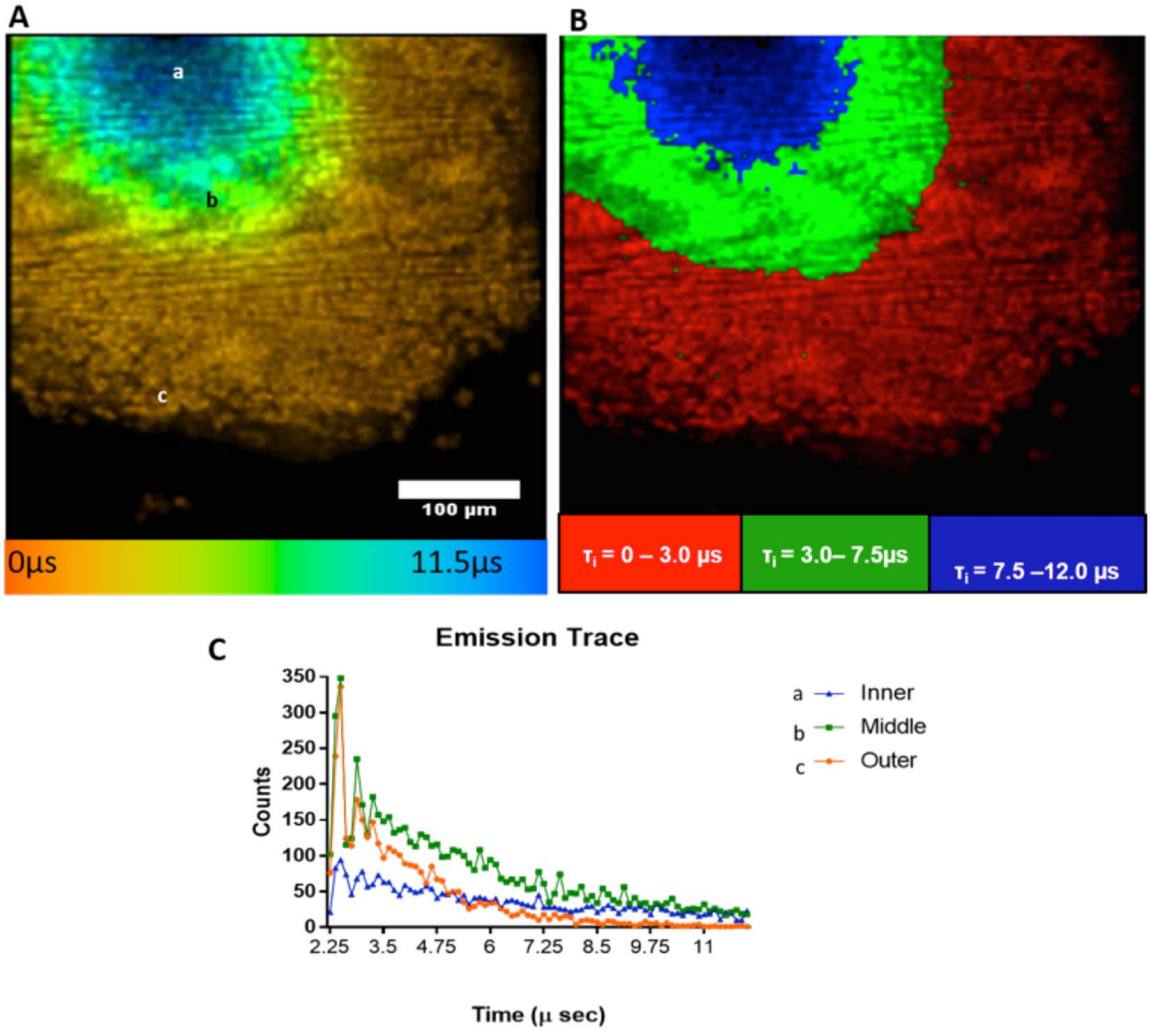
Lifetime distribution of PtL^s^Cl (100 µM, 12 hours) in melanoma MCTS using one-photon PLIM. PtL^s^Cl lifetime distribution across spheroid represented as: (**A**) Continuous rainbow scale, range: 0–11.5 µs. (**B**) Discrete colours, reveals three distinct spheroid regions: an outer proliferative rim (red, 0–3.0 µs), an intermediate necrotic region (green, 3.0–7.5 µs) and an inner necrotic core (blue, 7.5–12.0 µs). (Magnification 40x scale bar = 100 µm). (**C**) Emission decay observed in inner (a), middle (b) and outer (a) area of spheroid showed short-lived emission in outer area and a longer emission in inner area.

**Figure 4:**
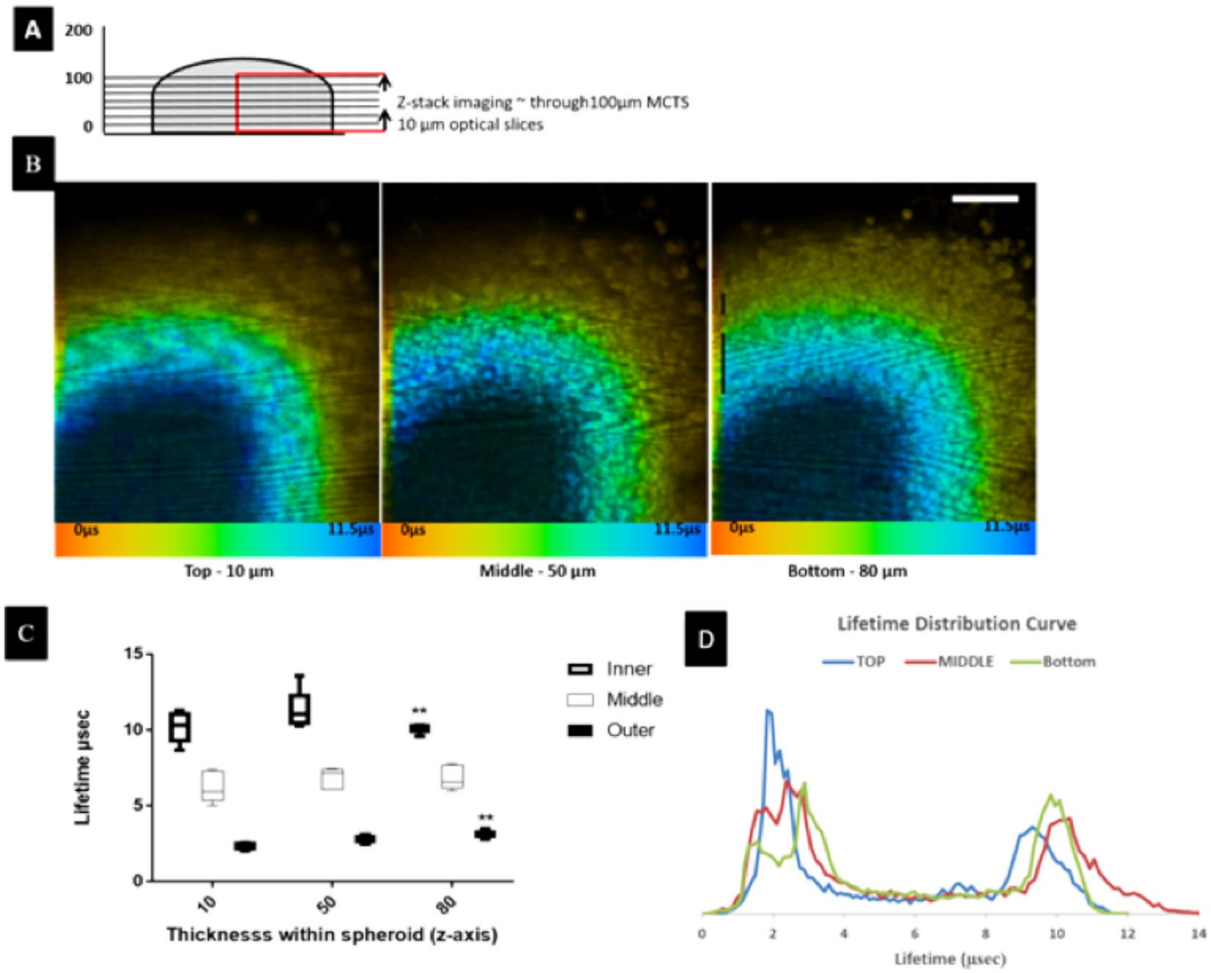
Z-stack lifetime distribution of PtL^s^Cl in melanoma MCTS using one-photon PLIM. (**A**) representation of a MCTS area (red) imaged using one-photon PLIM across ~100 µm. (**B**) Lifetime distribution at different depth (z-stack, Scale bar 200 µm) of MCTS. (**C**) Lifetime values within three different regions (outer, middle, and inner) of MCTS were measured (15 lifetime values, from each data set taken at different depths). Average lifetime values are used to plot lifetime differences within regions of spheroid. Lifetime of central necrotic core becomes shorter towards the periphery of the spheroid. Results shown are mean ± SD (n = 5) (**) denotes statistically significant different when outer (proliferative area) was compared with inner necrotic area. (**D**) Lifetime distribution histogram of spheroid with PtL^s^Cl.

A Becker & Hickl (GmbH) time correlated single photon counting (TCSPC) module, coupled to a confocal microscope and a photomultiplier, was used for photon detection. In a one-photon set-up the pixel clock, which controls the scan head of the microscope, acts as the trigger for the TCSPC module enabling the emission decay of each pixel in the 256 × 256 array to be analysed independently of one another. To construct lifetime maps, decay traces for all pixels across the 256 × 256 array (or those selected as a region of interest) were fitted to a double exponential model, with a bin (averaging parameter) of 2, and τ_i_ was plotted as a rainbow chart (and as discrete colours) to show the lifetime variation (and distinct regions) across the melanoma spheroids. The decay kinetics of PtL^s^Cl emission can be readily quantified in different cells and in different regions of a cell and tissue (Figs 3 and 4 and SI 3). A bi-exponential fit was reported previously both in cells and tissue^4, 17^. Hereby, it was observed that the temporal decay in melanoma spheroid fits well to a bi-exponential function. This bi-exponential decay kinetics is probably a simplest reflection of the complex distribution of tissue microenvironment and binding modes anticipated for the PtL^s^Cl with the tissue.

PLIM revealed that the emission lifetime of PtL^s^Cl in MCTS could be classified in to three distinct ranges; the shortest lifetime (ca. 0–4 µs, red, discrete scale) were observed at the outer edges of the MCTS, which increased to c.a 4–6.5 µs (green) then ca. 6.5–12 µs (blue) with increased proximity to the centre of the spheroid (decay best fit to a double exponential in all cases) (Figs 3 and 4). These distinct regions correlate clearly with Hypoxyprobe™ and Ki-67 staining, revealing the presence of an outer proliferative rim (τ = 2.14 ± 0.2 µs), an inner (or intermediate) hypoxic region (τ = 6.7 ± 0.5 µs) and a central necrotic core (τ = 9.6 ± 0.4 µs) at a depth of 80 µm. Furthermore, emission lifetime observed at the proliferative rim where cells have a good exchange of nutrients and waste products, match those observed in the cytosol of single cell monolayers (see SI, Figure S3). This distribution in emission lifetime and therefore oxygen concentration across the 3D spheroid is indicative of a solid microenvironment where physical structures for diffusion-limited conditions exist. Distribution of PtL^s^Cl lifetimes in monolayers of human HBL melanoma and HaCat keratinocyte cells also followed the previously observed trend of emission lifetime in the nucleoli > nucleus > cytoplasm, (see SI, Table 1).

#### PLIM lifetime maps

This is the first example of real-time oxygen mapping across a 3D human spheroid model using one-photon PLIM imaging, at high resolution. The data has provided a spatial-lifetime across the section of the spheroid, with enhanced resolution in the x, y plane revealing a clear cellular structure at the edges of the spheroid where the cells are intact and proliferating normally, and a lack of structure at the centre of the spheroid where there is mostly cell debris. Z-stacking data obtained by PLIM (Fig. 4) did reveal an increase in emission lifetime with increasing depth at the centre of the spheroid, indicative of an oxygen deficient core. However, this was limited to 100 µm due to relative penetration and high light scattering thereafter where no clear cellular structure could be observed.

## Discussion

Intracellular oxygen concentrations play a critical role in many physiological and pathological processes^35, 36^. The measurement of oxygen is important for assessing cellular activity and in clinical diagnosis, tumour chemo-sensitivity, in pharmacological drug screening and for environmental toxicant screening. Furthermore, changes in oxygen levels during tumour development can form the basis for the identification of neoplastically-transformed cells as micro-tumours^26, 27^.

PtL^s^Cl lifetime mapping using PLIM on MCTS of different sizes and growth stages cumulatively showed large spheroids having a significant lifetime-oxygen gradient, while smaller and earlier growth stage spheroids showed no such gradient. This heterogeneity in lifetime was dependent on spheroid culture conditions. Finally, pixel-by-pixel measurements of lifetime in these three distinct areas allowed demographic quantification of average lifetimes values in spheroids across the z-axis, illustrating a sensitive, real-time, quantification technique.

Moreover, we observed a diffusion limited cell permeability property of the small molecule PtL^s^Cl type, and diffusion-limited properties through a multi-layered tumour spheroid, where a longer incubation time using the same concentration as for single cell imaging was required. This property, in combination with a high emission quantum yield and moderately long emission lifetime, which is strongly oxygen sensitive, offers a simple imaging procedure. This has potential in terms of PLIM acquisition times where subsequent excitation pluses must be separated by at least 4 to 5 times the length of the emission lifetime of the phosphorescent probe. Following on from this study we aim to investigate the full optical slicing ability of two-photon PLIM with PtL^s^Cl, to assess applicability (via topical application) in keratinized cancer skin models for broader clinical applications of melanoma diagnosis.

The present study demonstrates a novel approach for the detection of relative oxygen levels in a melanoma tumour tissue mass, based on the ability to combine PLIM and single-photon excitation in combination with an oxygen sensitive platinum label. Given that solid tumours display varied oxygen levels, this characteristic can be exploited to develop new diagnostic tools to determine and exploit these variations and we therefore present this as a novel optical method. The data shows one of the first examples of non-invasive oxygen mapping across a melanoma tumour spheroid using one-photon PLIM, revealing a variation in lifetime that correlated with oxygen concentration. These measurements were quantitative and enabled real time oxygen mapping with enhanced spatial resolution.

There is experimental data that suggests that hypoxic cells within tumour microenvironments are less sensitive to chemotherapy and radiotherapy. In addition, it has been suggested that tumour hypoxia may also relate to malignant progression^37, 38^. Our PtL^s^Cl probe along with confocal PLIM provides information that may be useful in selecting patients who would benefit from new hypoxic- targeted therapy^39^. We suggest that the development of a non-invasive 3D mapping of the tumour microenvironment with high resolution will open up new avenues for therapeutic interventions. For example, investigating the hypoxic environment of a tumour *in vivo* would be of value in the surgical management of melanoma or in the use of high precision irradiance modulation to achieve selective dose escalation in chemo/radio resistant hypoxic cells within the tumour microenvironment. The study of this family of probes benefits from access to multiphoton microscopy.

Parallel developments in dyes and imaging technology are also being studied^40^. The direction of research is that it should prove possible to use fibre optic probes for use in the clinic, which would allow the interrogation of tumours within the skin up to a depth of 0.5 mm, sufficient to detect surface, deep and metastasising melanoma leaving the primary tumour. Thus, one can excite the compound with 400 nm light, and emission is such that fibre optic excitation with long or short wave light will still lead to a desired measurement. We therefore suggest PLIM incorporated with PtL^s^Cl probes may be developed as a technology and optical tool for the detection of both physiological and pathological oxygen levels in live tumour tissue mass, with the potential for broader clinical application.

## Methods

### PtL^s^Cl probe

The PtL^s^Cl probe was synthesised as described by us previously^19^. Other imaging dyes and stains were purchased from commercial suppliers (as indicated in the SI) and used as per the suppliers instructions, all other reagents were purchased from Sigma Aldrich (Poole, Dorset, U.K.).

### Human melanoma cells

The human melanoma cell line C8161 was assessed for its ability to form and grow as an MCTS, using the liquid overlay method previously described^16^. Briefly, 100 µL of cells (1.2 × 10^5^ per mL) were added to each well of a 96-well plate previously coated with type V agarose (1.5% (w/v)) in the growth medium (EMEM). The cells were incubated at 37 °C, 5% CO_2_ and monitored overtime for spheroid production. Cell culture medium was refreshed every 2–3 days by replacing 100 µL with fresh medium. MCTS was imaged every day for 12 days using phase contrast light microscopy to detect spheroid formation (see SI, Fig. 1). Experiments were performed in triplicate. Analysis of MCTS using haemotoxylin & eosin, Ki-67 and Hypoxyprobe™ are detailed in the supplementary information, along with details of individual cell cultures and 2D co-cultures. Spheroid growth and radius of the specific areas within spheroids was measured using ImageJ software (version 1.4 G). The mean values were calculated and sample statistical distribution was derived from a standard error, which was calculated using the formula: standard deviation/√ (count (range of number)).

### Melanoma staining with PtL^s^Cl

Staining with PtL^s^CL and imaging via one-photon PLIM is briefly described below for clarity, full details of staining spheroids with PtL^s^CL can be found in the SI. To prepare melanoma spheroids for PLIM microscopy, spheroids were removed after 6–8 days in culture and transferred to 35mm glass bottomed dishes (3–4 MCTS in each dish) and allowed to settle overnight at 37 °C, 5% (v/v) CO_2_ in full EMEM medium. Spheroids were then incubated with PtL^s^Cl 100 µM for 12 hours (0.5% DMSO, in PBS). Before imaging, spheroids were washed with PBS (x3) and then immersed in PBS.

### Phosphorescence Lifetime Image Mapping

Measurements were performed at the Central Laser Facility, Rutherford Appleton Laboratory, STFC, Oxford, UK. The set-up comprised a Nikon microscope connected to a Becker and Hickl DCS-120 confocal scanning FLIM system and a Becker and Hickl 405 nm diode laser (BDL-405-SMC). Excitation and emission filters used were λ_ex_ = 405 nm, λ_em_ = 500–550 nm, respectively. The diode laser was ‘on/off’ controlled for PLIM imaging by the Becker and Hickl FLIM module. The lifetime imaging window was set to 32 µs, which included a laser on rise time of 2 μs. The field of view was made up of a 128 × 128-pixel array.

### Lifetime map analysis

Emission lifetime maps were constructed using Becker and Hickl SPCImage software (version 3.89). Decay kinetics were best fit to a double exponential approximation on a pixel-by-pixel basis. Pixel binning and threshold function were kept constant for all measurements. Lifetime data collected on single cells were taken from three independent regions of interest for each cell line. PtL^s^Cl phosphorescence lifetime was measured at three intercellular positions: (1) nucleus, (2) cytoplasm and (3) nucleoli with 14 independent sample points for each region, averaged and plotted against standard deviation (Figure SI3). PtL^s^Cl lifetime was measured across the spheroids (100 µm across and 20 µm apart) and lifetime emission images were stacked together using ImageJ software (version 1.4 G) for a volume 3D image of the whole construct. Furthermore, PtL^s^Cl phosphorescence lifetime values were measured at three regions of the spheroid: (1) outer; (2) middle and (3) central, which were averaged and plotted (n = 5 independent areas using xy coordinates for each spheroid area) against standard deviation. In order to check for any statistically significant differences, a paired t-test was performed. Results with p-values of ≤0.05 were considered statistically significant. All data was analysed using Graphpad Prism software, version 6.0 (Figs 3 and 4).

## Electronic supplementary material

Supplementary information
