# Supplementary material for: Oxygen Mapping of Melanoma Spheroids using Small Molecule Platinum Probe and Phosphorescence Lifetime Imaging Microscopy
Source: Sci Rep. 2017 Sep 6;7:10743. doi: 10.1038/s41598-017-11153-9 (PMC5587740; doi:10.1038/s41598-017-11153-9)
Supplement: Supplementary file 1 — Supplementary information [file 41598_2017_11153_MOESM1_ESM.pdf]

Supplementary information

Oxygen Mapping of Melanoma Spheroids using Small Molecule Platinum Probe and Phosphorescence Lifetime Imaging Microscopy

Ahtasham Raza[1], Helen E. Colley[2], Elizabeth Baggaley[3], Igor V. Sazanovich[4], Nicola H. Green[5], Julia A. Weinstein[6], Stanley W. Botchway[7], Sheila MacNeil[8], and John W. Haycock[9*]

[1,5,8,9] Materials Science & Engineering, University of Sheffield, Sheffield, S37HQ, UK.
[2] School of Clinical Dentistry, University of Sheffield, Sheffield, S10 2TA, UK.
[3,4,6] Department of Chemistry, University of Sheffield, Sheffield, S3 7HF, UK.
[7] Research Complex at Harwell, STFC Rutherford Appleton Laboratory, Oxford, OX11 0QX, UK. [*]j.w.haycock@sheffield.ac.uk

Keywords

Oxygen Fluorescence lifetime imaging microscopy, phosphorescence lifetime, two-photon imaging microscopy, spheroids, tissue engineering, oxygen.

Contents

1. Materials (Page: 1)
2. Human Melanoma and Keratinocyte cell culture (Page: 2)
3. Staining of 2D live cell cultures using PtL^sCl (Page: 2)
4. Human melanoma MCTS formation (Page: 2)
5. Human melanoma MCTS growth analysis (Page: 2-3)
6. Histology of human melanoma MCTS (Page: 3)
7. Analysis of spheroid hypoxia by Ki-67 (Page: 3)
8. Analysis of spheroid hypoxia by Hypoxyprobe™-1 (Page: 3)
9. PtL^sCl staining across melanoma spheroids (Page: 3-4)
10. In vitro emission spectra of PtL^sCl across melanoma spheroids (Page:4)
11. Additional Figures (Page: 5–8)

1. Materials

The PtL^sCl complex was synthesized as described previously [1]. Other fluorescent dyes, stains and reagents were purchased from commercial suppliers, as indicated: CellTracker™ Green CMFDA (5-chloromethylfluorescein diacetate) and CellTracker™ Red (Life Technologies Ltd, Paisley, UK), 4',6-diamidino-2-phenylindole (DAPI, BD Bioscience, Oxford, UK), Hypoxyprobe ™ and Anti-hypoxyprobe monoclonal primary antibody (HPI Ltd, MA, USA). All other materials were purchased from Sigma-Aldrich (Poole, Dorset, UK) and used according to manufacturer's instructions, unless otherwise stated. Dako blocking solution (Dako Ltd, UK), Pronase (Fisher Scientific), ABC elite, AP staining kit and Vectasheild mounting medium with DAPI (Vector Labs Ltd, Peterborough, UK). Agarose, trypsin (Detroit, Michigan, USA), Dulbecco's modified Eagles medium (DMEM, Biosera, UK), nutrient Hams F10 medium, Eagles minimum essential medium (EMEM), Roswell Park Memorial Institute medium (RPMI), penicillin, streptomycin, amphotericin-B (Fungizone®), non-essential amino acids (NEAA) (all from Gibco BRL, UK), fetal calf serum (FCS, GlobePharm, UK), new born calf serum (NBCS), phosphate buffer saline (Oxoid

Ltd, UK), Harris' haemtoxylin and eosin, Brij 35 (surfactant), bovine serum albumin (BSA). 35mm glass bottom dishes (20mm microwell no 1.5, coverglass 0.16-0.19 mm) (MatTek Corp, USA).

2. Human Melanoma and Keratinocyte cell culture

Three human melanoma cell lines (HBL, A375-SM and C8161) were used. The HBL cell line is derived from the lymph nodes of a metastatic modular melanoma (developed by Professor G. Ghanem, University of Brussels, Belgium) and was maintained in Hams F10 medium supplemented with 5% (v/v) FCS, 5% (v/v) NBCS, 2 μM L-glutamine, 100 IU/mL penicillin and 100 μg/mL streptomycin [6]. The A375-SM cell line was isolated from a lymph node metastasis of a melanoma patient (developed by Professor M.J. Humphries, University Manchester, UK). The C8161 line was derived from an abdominal wall metastasis from a recurrent malignant melanoma patient (Professor F. Meyskens, UC Irvine, USA) obtained via Dr. M. Edwards (University of Glasgow, UK). Both C8161 and A375-SM cells were maintained in EMEM medium with 10% (v/v) FCS, 2μM L-glutamine, 100 IU/mL penicillin and 100 μg/mL streptomycin and 1.2 μg/mL amphotericin B. The human keratinocyte cell line (HaCat) was established as an immortal adult line displaying a transformed phenotype with chromosomal abnormality, but without tumorigenicity [7]. HaCat cells were maintained in DMEM medium supplemented with 10% (v/v) FCS, 2μM L-glutamine, 100 IU/mL penicillin, 1.2 μg/mL streptomycin and 0.065 μg/mL amphotericin B. All cell lines were cultured at 37^0C/5% (v/v) CO_2 in a humidified atmosphere and passaged when 80% confluent.

3. Staining of 2D live cell cultures using PtL^sCl

Human keratinocytes (HaCat) and human melanoma HBL cells lines were seeded ($1x10^6$) separately on 35 mm glass bottomed dishes separately for 24 hours. Thereafter, medium was removed and cells were washed with PBS to remove any debris. Cells were labeled with 100 μM (PtL^sCl) (in 0.5%(v/v) DMSO/PBS) at 37°C for 5 minutes. Before analysis, monolayers were washed with PBS (x3) and kept immersed in PBS during imaging (λ_{ex} = 800nm, λ_{em} = 500-550nm) (Figure SI 3).

The cytotoxicity effect of PtL^sCl at a range of concentration (from 1 to 100 μM solution) with an incubation time of 5 minutes to 24 hours was previously tested on different mammalian cells and showed no significant reduction in cell viability [2, 3].

4. Human melanoma MCTS formation

The C8161 human melanoma cell line was used to form and grow as an MCTS, using the liquid overlay method previously described [4]. Briefly, 100 μL of cells (1.2×10^5 per ml) were added to each well of a 96-well plate previously coated with type V agarose (1.5% (w/v)) in growth medium (EMEM). The cells were incubated at 37°C, 5% CO2 and monitored overtime for spheroid production. Cell culture medium was refreshed every 2-3 days by replacing 100μL with fresh medium. MCTS was imaged every day for 12 days using phase contrast light microscopy to detect spheroid formation. Experiments were performed in triplicate.

5. Human melanoma MCTS growth analysis

As described in the Results, only C8161 melanoma cells formed consistent spheroids and thus further experiments were conducted using this melanoma cell type. MCTS formation and growth was monitored

by phase contrast light microscopy using a Zeiss Axiovert 200M inverted epifluorescence microscope fitted with an AxioCam digital camera (Nikon). Spheroid diameters were determined by image analysis every second day for 10 days using Axiovision 4.6 software (Imaging Associates Limited, Bicester, UK). A minimum of n=63 images were used for each time point (2, 4, 6, 8, 10, 12 days in culture). All experiments were performed in triplicate (Figure SI 1).

6. Histology of human melanoma MCTS

C8161 melanoma spheroids were fixed at each time point in 10% neutral buffered formalin solution (Sigma) for 24 hours, washed twice in PBS (for 5 minutes) and transferred to round bottomed cryovials (VWR). Excess PBS was removed before the addition of 100 μL 1% agarose (type V dissolved in 10 % (v/v) neutral buffered formalin) to the bottom of the each cryovial to embed the MCTS. Agarose embedded fixed MCTS were removed wax embedded, sectioned and H&E stained (Figure 2, A-B).

7. Analysis of spheroid hypoxia by Ki-67

To reveal proliferative cells within human melanoma spheroid model Ki-67 immuno-staining was performed. Briefly, spheroid samples sectioned at 4μm were de-waxed with xylene and then rehydrated through a series of alcohol solutions. Sections were immersed in H_2O_2 (3% in ethanol) solution to quench endogenous peroxidise activity. The sections were microwaved for 10 min (10mM citrate buffer) for antigen retrieval. After blocking samples with rabbit serum for 30 min at 37°C, sections were incubated with a primary antibody Ki-67 (rabbit polyclonal, Dako, Copenhagen, Denmark) for 60 min at 37°C. Biotinylated secondary Ab and streptavidin-HRP was later incubated for 30 min at 37°C (Vectastatin Elite ABC kit, Vector laboratories, Peterborough, UK). Staining was visualized using 3,3'–diaminobenzidine chromagen substrate followed by counter-staining with haematoxylin (Figure 2, E).

8. Analysis of spheroid hypoxia by Hypoxyprobe™-1

For immunohistochemistry detection of hypoxic regions within the MCTS, Hypoxyprobe™-1 (Chemicon Int, USA) at a final concentration of 170 μM (diluted in serum free medium) was added to each MCTS for 4 hours, washed with PBS (x3) and fixed in 3.7% (w/v) formaldehyde overnight. Samples were then processed for histology as above. MCTS sections (4 μm) were de-waxed in xylene and rehydrated in a series of ethanol solutions, washed with 0.2% (w/v) nonionic detergent (Briji 35 in deionized water) for 2 minutes followed by another wash with 0.2% (w/v) detergent (Briji 35 in PBS). Sections were neutralized for endogenous peroxidase using 3% (v/v) hydrogen peroxide in distilled water for 5 minutes. Antigen retrieval was conducted using 0.01% (w/v) Pronase (40 minutes), followed by protein blocking in Dako Blocking Solution (Dako UK, Ltd) for 5 min at room temperature. Samples were incubated with anti-hypoxyprobe™ mouse monoclonal antibody (1/50 (v/v) dilution in PBS with 0.1% BSA) for 40 minutes, washed with 0.2% (w/v) Brij35 in PBS for 5 minutes and incubated with biotinylated horse anti-mouse IgG secondary antibody (30 minutes, 1/500 (v/v) dilution) followed by another wash (0.2% (w/v) Brij 35 in PBS) for 5 minutes. Samples were then incubated with Vectastain® ABC-AP blocking reagent for 30 minutes, washed with 0.2% (w/v) Brij 35 in PBS for 5 minutes and incubated in alkaline phosphate substrate solution (Vector Red AP substrate kit®) for 15 minutes. Finally, samples were washed with tap water and counter-stained with 300nM DAPI solution and DPX mounted before imaging (Figure 2,C-D).

9. PtL^{s}Cl staining across melanoma spheroids

To measure PtL^{s}Cl distribution, spheroids were removed after 6-8 days in culture and transferred to 35mm glass bottomed dishes (3-4 MCTS in each dish) and allowed to settle overnight at 37°C, 5% (v/v) CO_2. Spheroids were then incubated with PtL^{s}Cl using 100 μM, 200 μM and 600 μM final concentrations for 1 hour or 12 hours. Before analysis, spheroids were washed with PBS (x3) and immersed in PBS during two-photon excitation (λ_{ex} = 800nm, λ_{em} = 500-550nm). Imaging was performed using a Zeiss LSM510 META upright confocal microscope, connected to a two photon class IV tuneable Ti-sapphire laser (Chameleon FD900, Coherent UK) and an Achroplan water dipping objective 10X lens (WD 3.1 mm, NA 0.3) was used to image PtL^{s}Cl emission through the spheroid. Optical slices were taken 10 μm apart to create a 3D z-stack construct using a frame size of 512 x 512, laser power (17 mW) and detector gain were kept constant for all samples (SI 2, A-D).

10. In vitro emission spectra of PtL^{s}Cl across melanoma spheroids

UV-vis spectrometer (Acton 275) and a CCD detector (Andor iDUS) attached to the microscopy port was used to study the emission spectra within spheroids to confirm complete penetration of the PtL^{s}Cl compound (emission is in the range 490–550nm). Spheroids were incubated with either PtL^{s}Cl for 12 hours or with no probe in PBS. The emission spectra were taken across 200 μm of sample with 10μm section sampling and each experiment performed in triplicate (SI 2, E).

10. Supplementary Figures

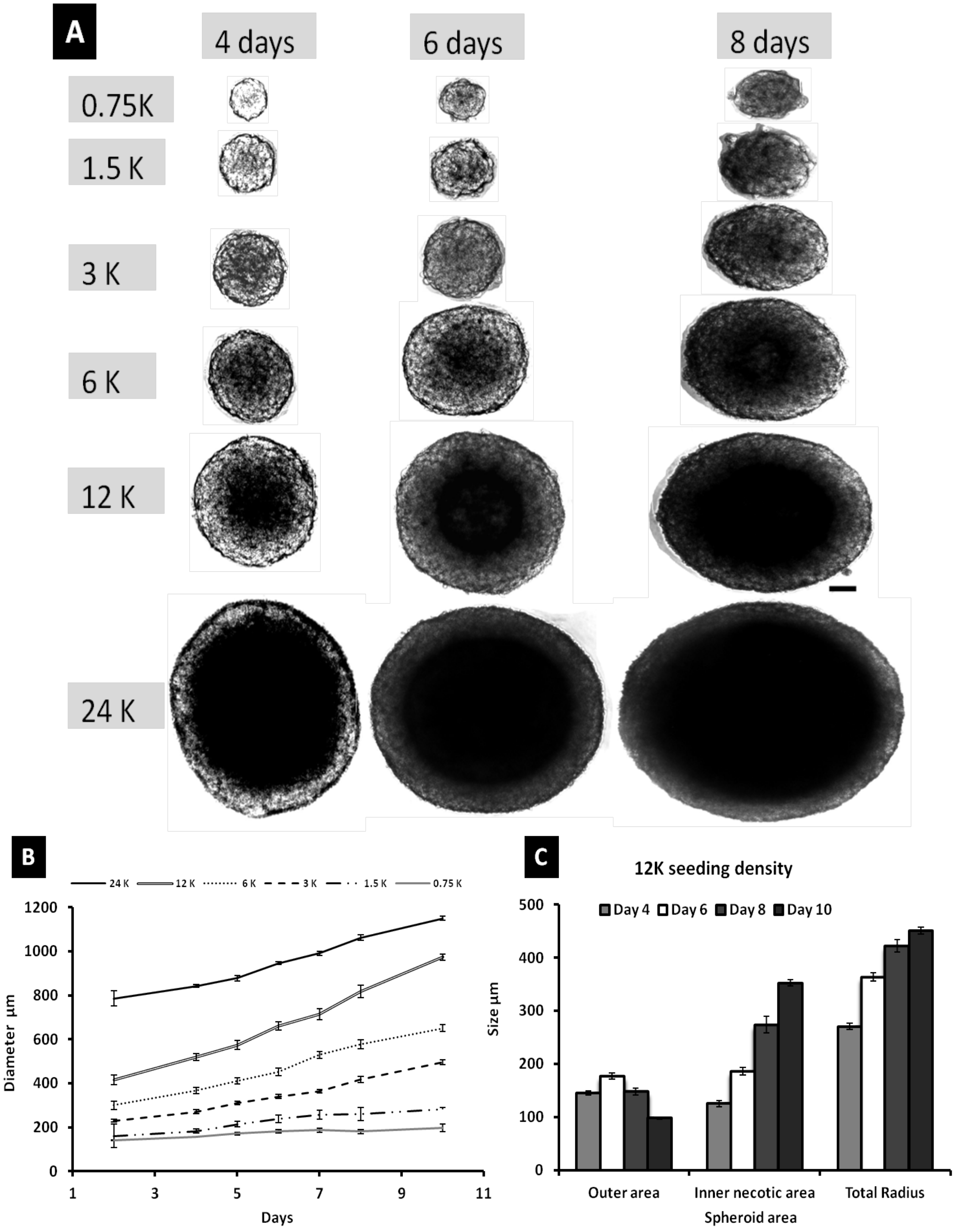

Figure S1: C8161 MCTS growth at different initial seeding density. (A) C8161 cells were seeded at 6 different concentrations 750 (0.75K), 1500 (1.5K), 3000 (3K), 6000 (6K), 12000 (12K), and 24000 (24K). (B) Cellular spheroid growth curve, where MCTS diameters measured was plotted against days in culture (C) Measurement of necrotic and outer area (proliferative) of C8161 MCTS at 12K initial seeding density. Images taken by phase contrast light microscopy (Magnification 5X, scale bar = 100 µm, error bars with standard error (n=10)).

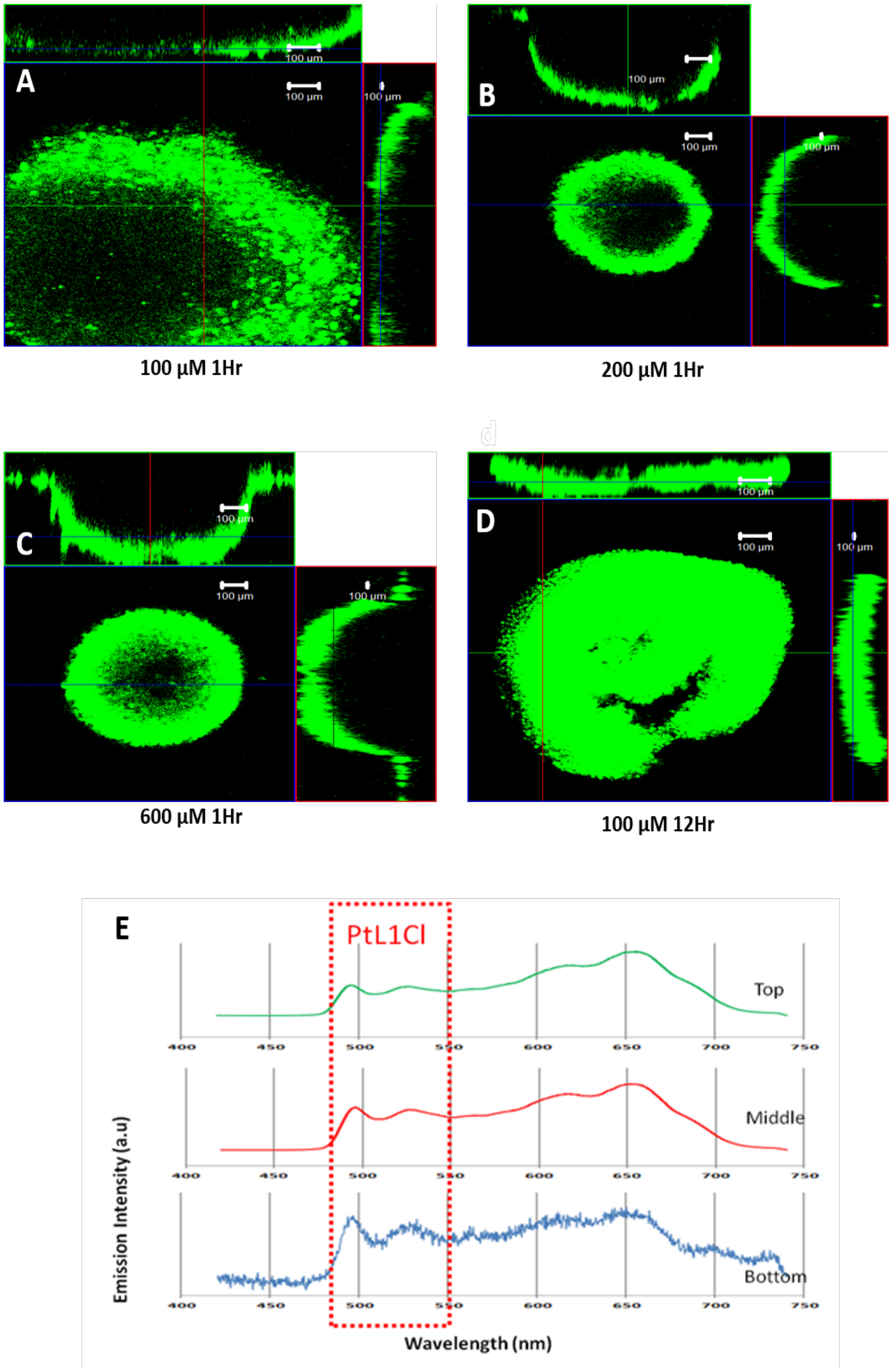

Figure S2: Steady-state emission of PtL^{s}Cl in melanoma C8161 MCTS using 2-photon microscopy. C8161 spheroid cultivated at day 6 (initial seeding density of 12,000 cells/well), placed on a well plate (overnight) in medium. Spheroids diameter was noticed to be in range of 500-600 µm after overnight settlement. Labelled with PtL^{s}Cl compound (a) 100 µM solution (b) 200µM solution (c) 600 µM solution incubated for 1 hour and washed with PBSx3. (d) Spheroid labelled with 100µM PtL^{s}Cl solution, incubated for 12 hours. Z-stack 2PE microscopy images taken every 10µm (λ_{ex} = 800 nm, λ_{em} = 500-550 nm). 2PE microscopic images were taken. PtL^{s}Cl compound penetration depth from top calculated as (a) 40µm (b) 100µm and (c) 130µm. Notice the penetration of PtL^{s}Cl through the whole thickness of spheroid (d). (Magnification 10X, scale bar = 100µm). (E) The emission spectrum of PtL^{s}Cl (12 hour incubation) obtained within MCTS (6day) at different depths confirmed complete intra-spheroid distribution of PtL^{s}Cl (emission is in the range 490–550nm).

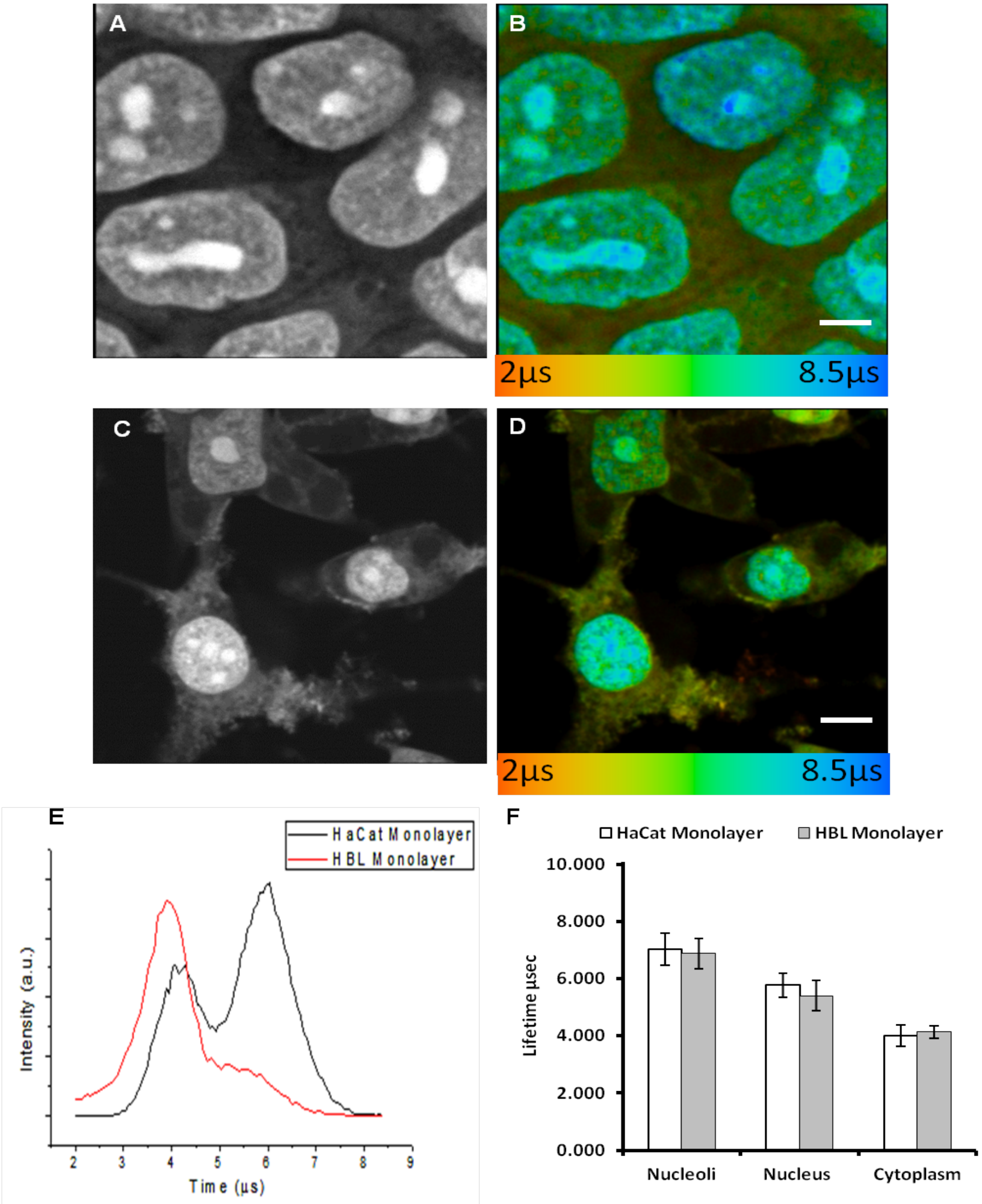

Figure S3: Phosphorescence Lifetime distribution of PtL^{S}Cl in Live cells. Live human keratinocyte cells (HaCat) and human melanoma cells (HBL) were labelled with PtL^{S}Cl (5 minutes, 37°C, 100 µM). Two-photon (λ_{ex} = 800 nm) PLIM Images: Intensity (A) and lifetime distribution (B) of PtL^{S}Cl in HaCat cells. Intensity (C) and lifetime distribution (D) of PtL^{S}Cl in HBL cells. Rainbow colour range: 2 - 8.5 µs (E) Lifetime distribution represents histogram for HaCat cells (red trace) and HBL cells (blue trace). The lifetime distribution monitored in HaCat and HBL range from 2 -8.5 µsec. (F) Lifetime values within three different regions (nucleoli, nucleus, cytoplasm) of HaCat and HBL cells are measured (15 lifetime values from each data file). Average lifetime values were used to plot lifetimes differences within regions of each cell line. (Magnification 40X, scale bar = 5 µm).

	$PtL^{s}Cl$ Lifetime (mean values / µs)		
**Sample**	***Nucleoli***	***Nucleus***	***Cytoplasm***
HaCat monolayer	7.0 (± 0.6)	5.8 (± 0.4)	4.0 (± 0.4)
HBL monolayer	6.9 (± 0.5)	5.4 (± 0.5)	4.0 (± 0.2)
HaCat and HBL co-culture	7.4 (± 0.5)	6.2 (± 0.4)	5.4 (± 0.5)
	Proliferative rim	***Hypoxic inner***	***Necrotic core***
C8161 melanoma MCTS (80um depth) 1photon-PLIM	2.1 (±0.2)	6.6 (±0.5)	9.6 (±0.4)

References

1. Williams, J.A.G., et al., *An Alternative Route to Highly Luminescent Platinum(II) Complexes: Cyclometalation with N∧C∧N-Coordinating Dipyridylbenzene Ligands.* Inorganic Chemistry, 2003. **42**(26): p. 8609-8611.
2. Botchway, S.W., et al., *Time-resolved and two-photon emission imaging microscopy of live cells with inert platinum complexes.* Proc Natl Acad Sci U S A, 2008. **105**(42): p. 16071-6.
3. Baggaley, E., et al., *Two-photon phosphorescence lifetime imaging of cells and tissues using a long-lived cyclometallated Npyridyl^Cphenyl^Npyridyl Pt(ii) complex.* RSC Advances, 2014. **4**(66): p. 35003-35008.
4. Carlsson, J. and J.M. Yuhas, *Liquid-overlay culture of cellular spheroids.* Recent Results Cancer Res, 1984. **95**: p. 1-23.
5. Baggaley, E., et al., *Long-lived metal complexes open up microsecond lifetime imaging microscopy under multiphoton excitation: from FLIM to PLIM and beyond.* Chemical Science, 2014. **5**(3): p. 879-886.
6. Eves, P., et al., *Melanoma invasion in reconstructed human skin is influenced by skin cells investigation of the role of proteolytic enzymes.* Clinical & Experimental Metastasis, 2003. **20**(8): p. 685-700.
7. Boukamp, P., et al., *Normal keratinization in a spontaneously immortalized aneuploid human keratinocyte cell line.* J Cell Biol, 1988. **106**(3): p. 761-71.
8. Papkovsky, D.B., *Methods in optical oxygen sensing: protocols and critical analyses*. Methods Enzymol, 2004. **381**: p. 715-35.
